# Glycogen distribution in the microwave‐fixed mouse brain reveals heterogeneous astrocytic patterns

**DOI:** 10.1002/glia.23020

**Published:** 2016-06-29

**Authors:** Yuki Oe, Otto Baba, Hitoshi Ashida, Kouichi C. Nakamura, Hajime Hirase

**Affiliations:** ^1^ Laboratory for Neuron‐Glia Circuitry, RIKEN Brain Science InstituteWakoSaitamaJapan; ^2^ Oral and Maxillofacial Anatomy, Graduate School of Oral Sciences, Tokushima UniversityTokushimaJapan; ^3^ Laboratory of Biochemistry Frontiers, Graduate School of Agricultural Science, Kobe UniversityHyogoJapan; ^4^ Department of Morphological Brain ScienceGraduate School of Medicine, Kyoto UniversityKyotoJapan; ^5^ Saitama University Brain Science InstituteSaitamaJapan

**Keywords:** astrocytes, focused microwave irradiation, glycogen, immunohistochemistry, aging

## Abstract

In the brain, glycogen metabolism has been implied in synaptic plasticity and learning, yet the distribution of this molecule has not been fully described. We investigated cerebral glycogen of the mouse by immunohistochemistry (IHC) using two monoclonal antibodies that have different affinities depending on the glycogen size. The use of focused microwave irradiation yielded well‐defined glycogen immunoreactive signals compared with the conventional periodic acid‐Schiff method. The IHC signals displayed a punctate distribution localized predominantly in astrocytic processes. Glycogen immunoreactivity (IR) was high in the hippocampus, striatum, cortex, and cerebellar molecular layer, whereas it was low in the white matter and most of the subcortical structures. Additionally, glycogen distribution in the hippocampal CA3‐CA1 and striatum had a ‘patchy’ appearance with glycogen‐rich and glycogen‐poor astrocytes appearing in alternation. The glycogen patches were more evident with large‐molecule glycogen in young adult mice but they were hardly observable in aged mice (1–2 years old). Our results reveal brain region‐dependent glycogen accumulation and possibly metabolic heterogeneity of astrocytes. GLIA 2016;64:1532–1545

## Introduction

Tissue glycogen is a substrate for short‐term storage of cellular energy in the body. Upon the activation of glycogen phosphorylases and debranching enzymes, phosphorylated glucose is restored from glycogen and utilized in energy metabolism. Glycogen storage and usage notably occur in the liver and muscle to maintain the blood glucose level and meet extra energy demand. Although in smaller amounts, glycogen is also stored in the brain (Brown and Ransom, 2007). The functional role of brain glycogen has been considered to be an on‐demand source of energy. The enzymatic breakdown of glycogen eventually produces pyruvate, a proportion of which is converted to lactate. According to the “lactate shuttle” hypothesis, astrocytic lactate is transferred to neurons to fuel the tricarboxylic acid cycle (Pellerin et al., 2007; Tsacopoulos and Magistretti, 1996). Importantly, a series of studies have shown that glycogenolysis and the resultant lactate transport play a key role in long‐term potentiation of synapses and the consolidation of memory in vertebrates (Gibbs et al., 2006; Newman et al., 2011; Suzuki et al., 2011).

Despite its importance in both energy metabolism and memory, characterization of brain glycogen distribution has not been fully addressed. The localization of glycogen has been shown in astrocytes by electron microscopy, by which techniques glycogen complexes are visualized as electron‐dense granules (Maxwell and Kruger, 1965; Cataldo and Broadwell, 1986a, 1986b, but see Sinadinos et al., 2014 for neuronal accumulation during aging or in Lafora's disease model mice). However, electron microscopy is limited to the subcellular level and not suitable for investigating in larger scales. Periodic acid‐Schiff (PAS) staining has been traditionally used to visualize glycogen in fixed tissues (McManus, 1948), yet PAS staining lacks specificity to glycogen because it also reacts with glycoproteins and other polysaccharides. Moreover, transcardiac perfusion fixation or acute dissection of the brain decreases the glycogen level, most likely due to anaerobic metabolism during the hypoxic states (Fiala et al., 2003; Kong et al., 2002).

**Figure 1:**
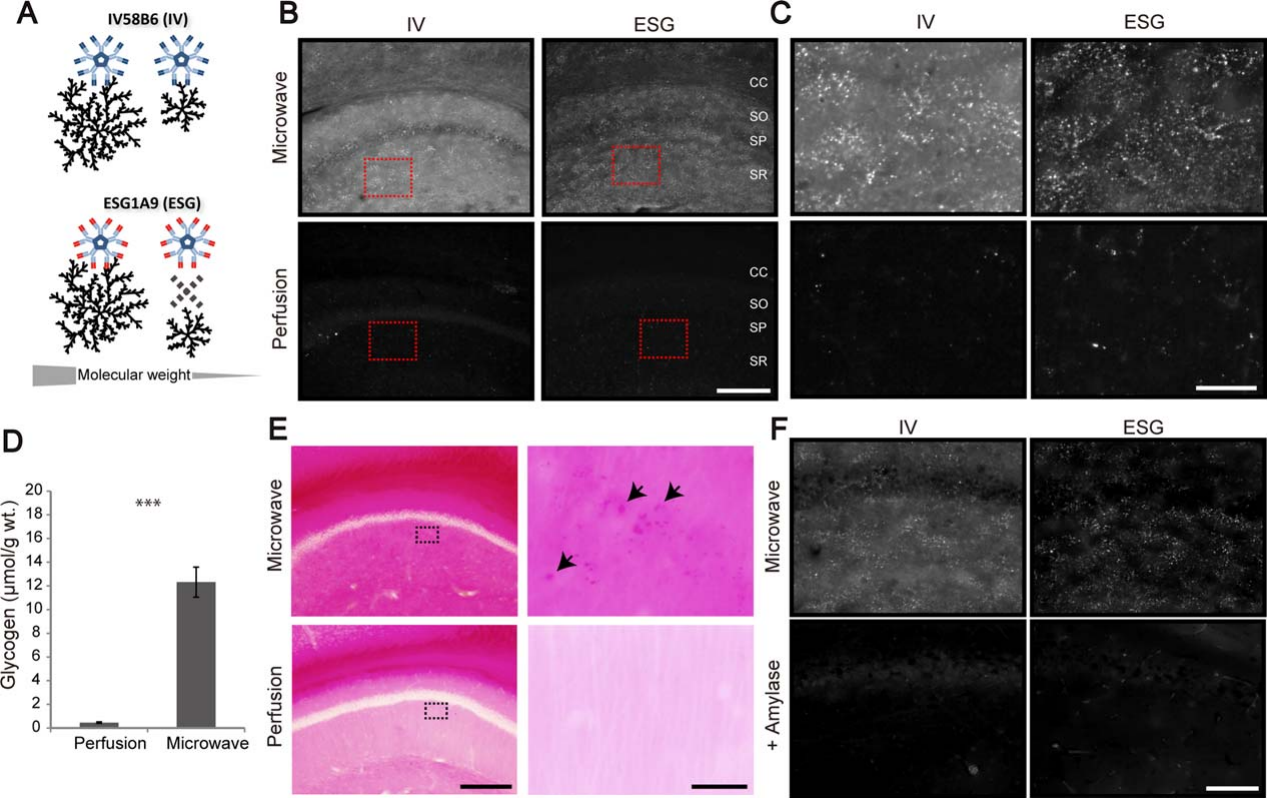
Glycogen IHC using IV58B6 and ESG1A9 antibodies. (**A**) Schematic illustration of antibody affinity to glycogen. (**B**) Comparison of glycogen IHC using two different euthanasia methods: focused microwave irradiation‐assisted fixation (see methods) *vs*. perfusion fixation. Both IV58B6 (IV) and ESG1A9 (ESG) antibodies were subjected to IHC of the mouse dorsal hippocampal CA1. CC *corpus callosum*, SO *stratum oriens*, SP *stratum pyramidale*, SR *stratum radiatum*. (**C**) Magnified views of glycogen IHC in CA1 *stratum radiatum* from doted red box in (**B**) with the same arrangement as in (B). (**D**) Comparison of whole brain glycogen content by biochemical assay. (mean ± SEM, *N* = 6, ***P < 0.001, unpaired t‐test). (**E**) PAS staining of microwave‐fixed (upper) and perfusion‐fixed (lower) hippocampal CA1 sections. Dotted regions are magnified in the right panels. Arrowheads points to glycogen‐like granules which appear as more intense spots in the PAS staining. (**F**) Amylase‐digested hippocampal CA1 sections are not labeled with either IV58B6 or ESG1A9. Scale bars: (B) 200 μm; (C) 50 μm; (E) 200 μm; (left) 20 μm (right); (F) 100 μm. [Color figure can be viewed in the online issue, which is available at wileyonlinelibrary.com.]

Focused microwave irradiation halts enzymatic activities by instantaneous heat induction and preserves the metabolic state of the brain (Kong et al., 2002; Sugiura et al., 2014). Here we took an immunohistochemical approach using microwave‐fixed brains to visualize glycogen distribution. We used two different monoclonal antibodies (IV58B6 and ESG1A9) that have distinguishable affinities to glycogen of different molecular sizes (Fig. 1A, Baba, 1993; Nakamura‐Tsuruta et al., 2012); Whereas IV58B6 has affinity to natural glycogen of various sizes, ESG1A9 preferentially binds to larger natural glycogen molecules (e.g., 15 MDa type III rabbit glycogen) over smaller glycogen molecules (e.g., 29 kDa enzymatically digested glycogen) (Nakamura‐Tsuruta et al., 2012). We find that relatively high amounts of glycogen are present in the hippocampus, cerebral cortex, striatum, and cerebellar cortex. Large molecule glycogens are preferentially found in the hippocampus and striatum and showed distinct patchy patterns that disappear in aged mice (1–2 years old).

## Materials and Methods

The procedures involving animal care, surgery and sample preparation were approved by the Animal Experimental Committee of RIKEN Brain Science Institute and performed in accordance with the guidelines of the Animal Experimental Committee of RIKEN Brain Science Institute.

### Brain Tissue Preparation

Young adult (postnatal 9–12 weeks) and aged (postnatal 1–2 years) C57BL/6 male mice were euthanized by focused microwave irradiation using a MMW‐05 microwave fixation system (Muromachi Kikai, Tokyo, Japan). All mice were raised on regular diet with 12/12‐h light/dark cycles and sacrificed at 6:00–8:00 p.m. (i.e., within 2 h before the end of diurnal period). Briefly, an unanesthetized mouse was confined in a specialized tubulous animal holder with a hollow wall filled with water (WJM‐24 or WJM‐28, Muromachi Kikai). Once the mouse proceeds to the tip of the holder tube, the animal's position is fixed by placing a plunger and the holder is then placed inside MMW‐05. High‐energy microwave (5 kW) was focused at the head of the mouse for 0.94–1.05 s. Immediately after focused microwave euthanasia, the brain was removed from the skull and incubated in fixative [4% paraformaldehyde in 0.1 M phosphate buffer (PB)] for overnight at 4°C. In some experiments, mice were fixed with a standard perfusion‐based method for comparison. Briefly, transcardiac perfusion was performed with 25 mL saline (0.9% NaCl solution) for 5 min, followed by 50 mL of fixative (as above) for 10 min. Perfusion‐fixed brains were also postfixed for overnight.

For experiments comparing aged and control young adult mice (i.e., Fig. 8), mice from both groups were confined in the animal holder immediately after brief anesthesia with 3% isoflurane. We waited for two to three minutes for recovery from anesthesia before focused microwave was applied as above. Recovery of anesthesia is favored to keep the posture for focused microwave irradiation. Such a procedure is needed because the holder tube diameter is relatively small for aged mice to enter voluntarily. Glycogen immunohistochemical staining is qualitatively similar between the unanesthetized and the briefly‐anesthetized methods, as shown in examples in Figs. 1 and 8, respectively.

### Histology

Two IgM‐type monoclonal antibodies for glycogen (ESG1A9 and IV58B6) were used in this study. The generation of the monoclonal antibodies has been described in prior publications (Baba, 1993; Nakamura‐Tsuruta et al., 2012). Brain sections of 60 μm thickness were prepared in PB using a microslicer (Pro‐7 Linear Slicer, DSK, Japan). After washing in phosphate‐buffered saline (PBS), the sections were incubated in PBS containing 0.1% Triton X‐100 and a combination of primary antibodies for 24 h at in 4°C while gently shaking. The concentrations of primary antibodies were as follows: ESG1A9 1:50, IV58B6 1:300, GFAP (AB7260, Abcam) 1:1000, Iba 1 (019‐19741, WAKO) 1:1000, NeuN (ABN78, EMD Millipore) 1:1000; denatured GFP (Nakamura et al., 2008) 1:300. The final antibody concentrations of ESG1A9 and IV58B6 corresponded to 15 µg/mL and 30 µg/mL, respectively. The sections were then washed three to five times in Tris‐buffered saline and incubated with fluorescent secondary antibodies (1:1,000 in PBS containing 0.1% Triton X‐100, Alexa Fluor 488 or 594, Life Technologies). Brain slices were mounted on slide glasses and coverslipped with PermaFluor mounting medium (Thermo Scientific). For the experiment of glycogen specificity by amylase, some slices were treated with amyloglucosidase (A7095, Sigma‐Aldrich) in the following condition. 10U amyloglucosidase were adjusted to pH = 5.0 in phthalate buffer, and treated for 24 h in 45°C, which is the optimal condition for amyloglucosidase activity. Then the slices were subjected to glycogen immunohistochemistry as above.

PAS staining was performed as described previously (Kong et al., 2002). Briefly, 60 μm‐sectioned slices were oxidized by a treatment with 0.5% periodic acid for 10 min, followed by incubation in saturated dimedone aqueous solution at 60°C for 30 min. Reaction with Schiff's reagent was conducted for 5 min at room temperature.

Cobalt‐enhanced cytochrome oxidase staining was performed using a standard method as described previously (Gulyas et al., 2006; Silverman and Tootell, 1987).

For YFP expression in sparse astrocytes, a single dose of tamoxifen (1 mg) was injected intraperitoneally in mice carrying GLAST‐CreERT (Mori et al., 2006) and Rosa26‐LSL‐EYFP (Madisen et al., 2010) knockin genomes 10 days before the mice were sacrificed. This amount of tamoxifen induced EYFP expression in approximately 50% of cortical astrocytes in a preliminary experiment.

### Biochemical Assay of Glycogen

Biochemical quantification of glycogen was performed by a commercial glycogen assay kit (K646‐100, BioVision). After microwave or perfusion fixation, the weight of a whole brain or brain tissue specimens was measured before homogenization. Homogenized samples were adjusted to have a concentration of 10 mg/200 µL in distilled water, followed by centrifugation by 13,000 rpm for 5 min. Supernatant and glycogen standard were transferred to a 96 well plate, followed by incubation with 1 µL hydrolysis enzyme mix for 30 min. Subsequently, the samples were incubated with 1 µL development enzyme mix and 0.3 µL OxiRed probe for 30 min in room temperature. The fluorescence intensity of samples was measured by a microplate reader (Thermo Scientific, Varioskan Flash; Ex/Em 535/587nm). After measurement, glycogen concentration was calculated from the calibration curve obtained by the glycogen standard. The final glycogen concentration was computed by subtracting the background value (the signal without hydrolysis enzyme mix).

### Image Acquisition and Analysis

Images of coronal or sagittal sections were acquired by a Keyence all‐in‐one microscope (BZ‐9000). 3D stacked images were constructed with a z step of 10 µm, using a 10× Nikon objective lens (Plan Apo NA = 0.45, Nikon). Brain areas of interest were determined manually according to the interactive atlas viewer (http://atlas.brain-map.org) for individual slices, followed by fluorescence intensity measurement. Obviously glycogen‐poor areas were excluded from analysis, for instance, the white matter (except for the corpus callosum), axon bundles, and principal cell body layers of the hippocampus.

High resolution images were acquired with a FV1000 laser scanning confocal microscope (Olympus). Cortical or hippocampal layers were observed with a 40× objective lens (UPlanSApo NA = 0.95, Olympus, 2.6 pixels per 1 µm^2^) with a z step of 1 µm. For analysis, each layer was identified according to NeuN IR with ImageJ software (http://imagej.nih.gov/ij/), followed by measurement of fluorescence intensity. 2D autocorrelations for images were computed by custom‐written MATLAB scripts (MathWorks); briefly an image was normalized so that each pixel represents the *Z*‐score. The normalized image was subjected to calculation of unbiased 2D crosscorrelogram. Glycogen immunofluorescence with each cell type marker was observed with a 60× objective lens (UPlanSApo NA = 1.2, Olympus, water immersion), 2× digital zoom (23 pixels/1 µm^2^), and a z step of 0.5 µm.

To compute the area fraction of glycogen IR, i.e., the proportion of glycogen‐immunoreactive area in a region of interest (ROI), fluorescent images of a cell type marker was binarized with a threshold of 30% of the maximum fluorescence intensity. The supra‐threshold area was set as the ROI. Each frame of the image stack (10 frames, totaling to 5 μm in depth from the surface of a specimen) was analyzed separately. Subcellular glycogen distribution of EYFP‐labeled astrocytes was observed with a 60× objective lens with 4× digital zoom up, yielding 385 pixels/1 µm^2^ resolution. For subcellular ROIs, somata and primary processes of astrocytes were manually selected. Fine processes were determined by masking out somata and primary processes from the binarized astrocytic structure. The number of glycogen particles and area fraction of glycogen were determined from binarized images of glycogen immunofluoresence with a threshold of 30% of the maximum fluorescence intensity in each ROI using ImageJ and MATLAB.

For comparisons of two groups, unpaired *t*‐test was used. For multiple group comparisons, one‐way ANOVA followed by the post hoc Tukey's honestly significant difference (HSD) test was used. Significance values were computed by R.

## Results

### Glycogen Visualization by Immunohistochemistry

Immunohistochemistry (IHC) of microwave‐fixed adult mouse brain sections with the pan‐glycogen IV58B6 (IV) and large glycogen‐preferring ESG1A9 (ESG) antibodies was performed. Figure 1B shows the distribution of immunoreactivity (IR) to the respective antibodies in the dorsal hippocampal CA1 region. Microwave fixation was compared with perfusion fixation for glycogen IR. For both antibodies, microwave‐fixed samples resulted in intense IR that appeared to reflect a cytoarchitectural pattern of the tissue, while perfusion‐fixed samples had substantially lower IR (magnified images of CA1 *stratum radiatum* in Fig. 1C). In addition to IR in neuropil, glycogen IR was seen in perivascular areas in microwave‐fixed brain tissue (Supporting Information Fig. 1). We note that longer permeabilization time with triton X100 was necessary for good staining with IV58B6 and ESG1A9 IgM antibodies, which are about five times larger than IgG antibodies (Supporting Information Fig. 2).

To confirm the preservation of glycogen after microwave fixation, biochemical assessment of glycogen was performed with perfusion‐fixed or microwave‐fixed whole brain samples (see Materials and Methods). Microwave fixation achieved superior preservation of brain glycogen to perfusion fixation (Fig. 1D; glycogen content: 0.46 ± 0.06 vs. 12.32 ± 1.27 µmol/g, *n* = 6 each, *P* < 0.001), which was consistent with the IHC.

Conventional PAS staining was also performed for comparison. As previously reported by Kong *et al*. (2002), PAS staining also tended to stain microwave‐fixed sections more intensively (Fig. 1E). Notably, PAS staining yielded signals more uniformly than the glycogen IHC. Nevertheless, intensely stained puncta scattered throughout the hippocampus were observable in the magnified view (Fig. 1E, upper right panels), resembling the punctate glycogen IR despite lower contrast than IHC (Fig. 3A, as examined in more detail later). The PAS staining intensity of perfusion‐fixed sections was weak and high‐intensity puncta were hardly observable, while background‐like signals remained to a significant degree. In particular, the white matter yielded intense signals even after perfusion fixation, which presumably reflect reactions with glycoproteins, proteoglycans, or other polysaccharides. The weak signals with IHC and PAS staining of perfusion‐fixed sections are most probably due to the consumption of glycogen during the anoxic condition caused by the transcardiac perfusion process.

To further verify glycogen IR in microwave‐fixed sections, immunofluorescence labeling was performed on amylase‐treated sections, in which glycogen had been enzymatically digested. The amylase‐treated sections yielded little IR with either antibody (Fig. 1F), whereas control sections that underwent the same incubation condition without amylase produced similar glycogen IR as in Fig. 1B,C. In summary, these results indicate that both IV58B6 and ESG1A9 antibodies are specific to glycogen and can be utilized for immunohistochemical visualization of glycogen in the brain.

### Brain‐Wide Distribution of Glycogen in the Brain

**Figure 2:**
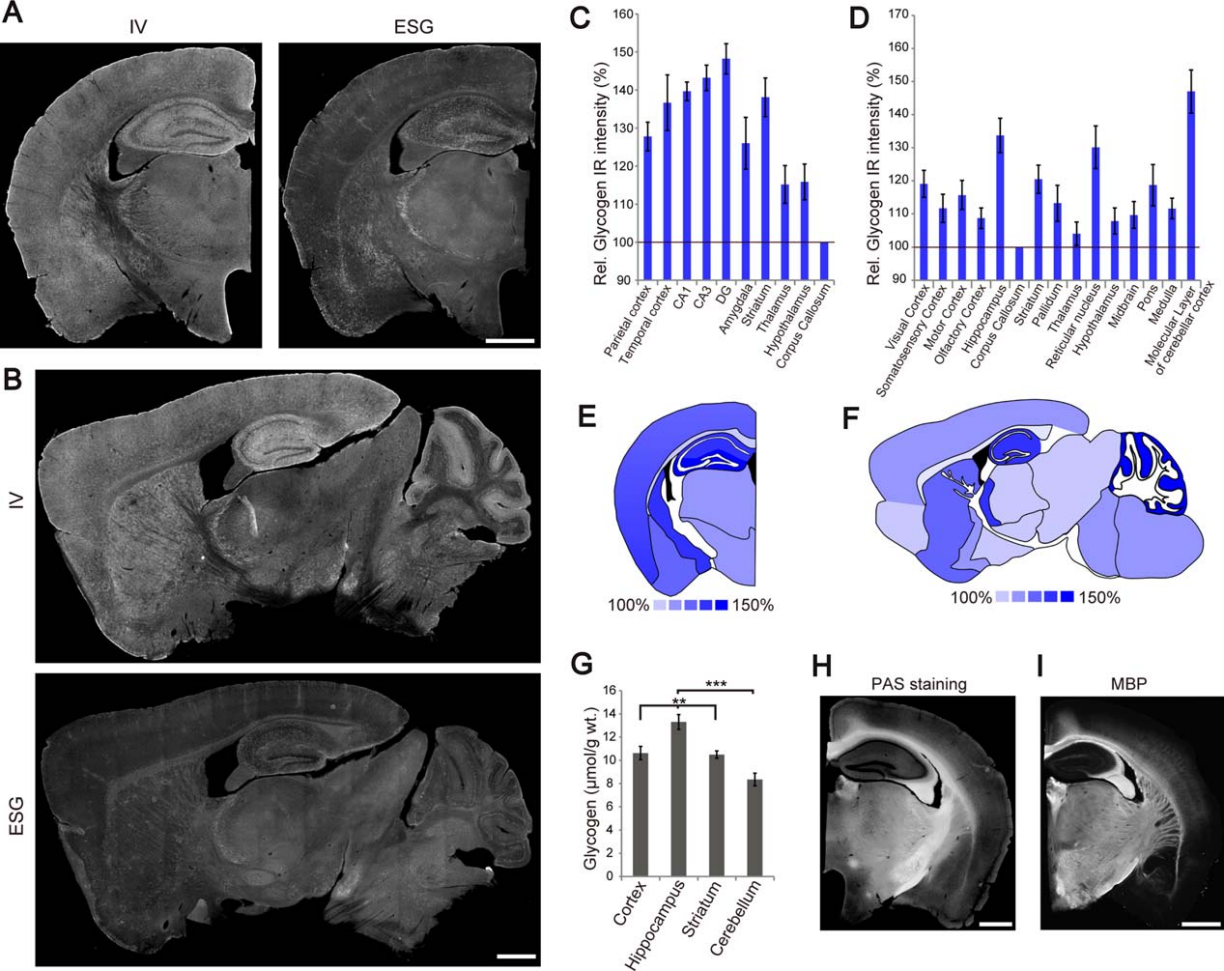
Brain‐wide distribution of glycogen in the brain. (**A**) Coronal sections of glycogen IHC by IV58B6 (left) and ESG1A9 (right). (**B**) Sagittal sections of glycogen IHC by IV58B6 (upper) and ESG1A9 (lower). Relative IV58B6 IR in various brain regions of the brain was evaluated for coronal (**C**) and sagittal (**D**) sections (mean ± SEM). These comparisons are pictorially represented in (**E**) and (**F**). (**G**) Glycogen assay for microwave‐fixed cortex, hippocampus, striatum, and cerebellum. Note that the cerebellar assay contains both the cerebellar cortex and white matter (mean ± SEM, *N* = 7, **P < 0.01, ***P < 0.001, one‐way ANOVA with Tukey HSD test). Fluorescence image of a PAS‐stained section (**H**) is compared with MBP IHC (**I**), which labels myelin. Scale bars: (A), (B), (H), and (I) 1 mm. [Color figure can be viewed in the online issue, which is available at wileyonlinelibrary.com.]

Having verified glycogen IHC using the IV58B6 and ESG1A9 antibodies in microwave‐fixed brains, we investigated the gross glycogen distribution in the mouse brain. Glycogen IHC resulted in wide‐spread labeling that had distinct patterns depending on the antibody and brain region. Coronal and sagittal sections are shown in Fig. 2A,B to demonstrate gross glycogen IR (other example images are shown in Supporting Information Figs. 3 and 4). Overall, visible labeling was achieved across wide areas of the brain with the pan‐glycogen IV58B6 antibody. In contrrast, the ESG1A9 antibody yielded generally lower IR, except in a few brain areas including the hippocampus and striatum.

First, we investigated glycogen distribution using IV58B6. We analyzed coronal sections that contain the dorsal hippocampus at the planes between 1.7 mm and 2.3 mm rostral to bregma. To quantify the relative intensity of glycogen IR, we normalized each section to the mean areal intensity of its corpus callosum (Fig. 2C, *N* = 9 mice). Similarly, sagittal sections containing the striatum, hippocampus, thalamus, and cerebellum were analyzed (Fig. 2D). Of the analyzed regions, the hippocampus, striatum, and cerebral cortex had higher IR than the subcortical structures. Of note, while the subcortical structures generally had low glycogen labeling, the thalamic reticular nucleus was labeled with as high intensity as the striatum. Moreover, the molecular layer of the cerebellar cortex showed conspicuously high IR, which presumably originates from the radial processes of Bergmann glia. The intensity maps of IV58B6 glycogen IHC in coronal and sagittal views are pictorially represented in Fig. 2E,F, respectively. Qualitative analysis for ESG1A9 localization is shown in Supporting Information Fig. 5. For biochemical confirmation, glycogen assay was performed on various brain regions. In agreement with the immunofluorescence results, the hippocampus had higher glycogen content than other areas (Fig. 2G). The apparent modest cerebellar glycogen content is most probably because the cerebellar specimens contained the white matter and other layers besides the molecular layer, which did not show high glycogen IHC signals (Fig. 2B).

We noticed that the corpus callosum had relatively low signals compared with the gray matter. This was in sharp contrast with PAS staining (Fig. 2H). Indeed, PAS staining yielded intense signals in areas with high densities of myelinated fibers such as the corpus callosum, hippocampal alveus and internal capsule, as illustrated by myelin basic protein (MBP) IHC (Fig. 2I). We supposed that this is a reflection of known reaction of PAS staining with glycoproteins and proteoglycans, which are abundant in the white matter.

### Glycogen is Localized in Astrocytes in the Forebrain

The differential patterns of glycogen IR by the two antibodies hinted that the distribution of glycogen molecules depends on the size of glycogen in addition to the brain region. To characterize size‐ and brain region‐dependent glycogen distribution, we next compared the detailed immunofluorescence view with the two antibodies using a confocal microscope in various brain regions including the cortex, hippocampus, striatum, and thalamus.

**Figure 3:**
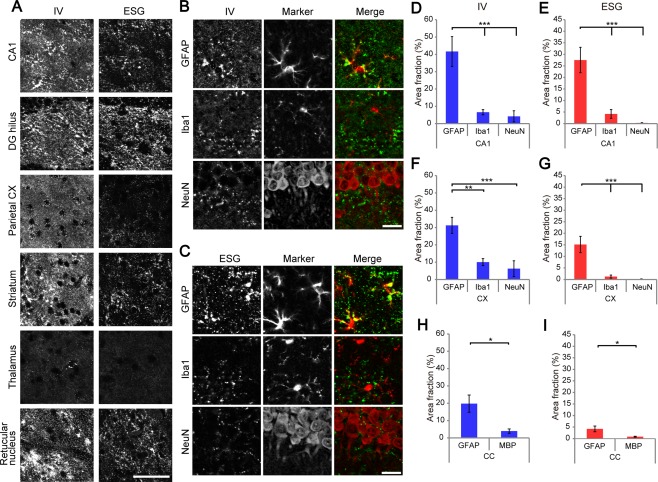
Punctate glycogen IR in various brain regions. (**A**) Confocal images of various brain regions showing glycogen IR with IV58B6 (left) and ESG1A9 (right) antibodies. (**B**) Double IHC of IV58B6 with cell‐type specific markers for astrocytes (GFAP), microglia (Iba1), or neurons (NeuN) in the CA1 *s.r*. (**C**) Double IHC of ESG1A9 with cell‐type specific markers, as in (B). Cell‐type dependent localization of glycogen IR by IV58B6 (**D**, **F**, and **H**) or ESG1A9 (**E**, **G**, and **I**) was examined by computing the area fraction of glycogen IR to a respective cell‐type specific marker (astrocyte, GFAP; microglia, Iba1; neuron, NeuN; oligodendrocyte, MBP) in the CA1 *s.r*. (D and E), cerebral cortex (F and G), and corpus callosum (H and I) (mean ± SEM, *N* = 7–11 animals, *n* = 140 images, *P < 0.05, **P < 0.01, ***P < 0.001, one‐way ANOVA with Tukey HSD test). Scale bars: (A) 60 μm; (B and C) 30 μm.

As shown in Fig. 3A, glycogen IHC displayed a punctate distribution in every examined area. Consistent with whole section imaging in Fig. 2, the hippocampus and striatum showed dense puncta distribution, while the thalamus and most of the subcortical structures were labeled sparsely except for the thalamic reticular nucleus. Furthermore, we noticed that where glycogen levels were high (e.g., hippocampus and striatum), large puncta (>0.5 µm in diameter) were observed. By contrast, smaller puncta (<0.5 µm) prevailed in low glycogen regions such as the thalamus. The cerebral cortex, where moderate IR was observed (Fig. 2), yielded both small and large puncta, although large puncta were not as prevalent as in the hippocampus or striatum. Intriguingly, ESG1A9 showed different immunoreactive profiles from IV58B6 in that large puncta are more emphasized in high glycogen areas. Moreover, low glycogen subcortical areas were hardly labeled. These results suggest that the presence of large‐molecule glycogen (labeled by ESG9A1) is a hallmark of glycogen‐rich area.

We next investigated the cellular and subcellular localization of glycogen in the hippocampal CA1 region by double immunofluorescence labeling using cell‐type specific markers. We used GFAP, Iba1, and NeuN antibodies for astrocyte, microglia, and neuron markers, respectively (Fig. 3B,C). Examination by confocal microscopy revealed dominant localization of glycogen with GFAP‐positive areas, whereas its localization with Iba1‐ or NeuN‐positive areas was minimal (Fig. 3D,E and Supporting Information Fig. 6). The predominant astrocytic localization was similar with IV58B6 and ESG1A9, in agreement with previous electron microscopic studies that reported electron‐dense glycogen particles in astrocytes (Calì et al., 2016; Cataldo and Broadwell, 1986a, 1986b; Fiala et al., 2003). Similar astrocytic localization was observed in other forebrain areas including the cerebral cortex (Fig. 3F,G) and corpus callosum (Fig. 3H,I).

**Figure 4:**
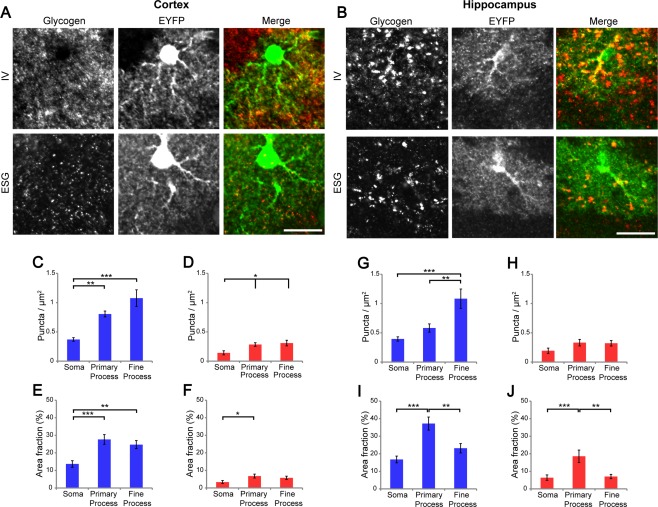
Subcellular distribution of glycogen in cortical and hippocampal astrocytes. (**A** and **B**) Confocal images of IV58B6 (upper row) and ESG1A9 (lower row) glycogen immunofluorescence with EYFP‐expressing astrocytes in Layer 1 of the parietal cortex (A) and hippocampus (B). EYFP expression in a sparse population of astrocytes was induced by tamoxifen administration to GLAST‐CreERT × Rosa26‐LSL‐EYFP mice. Scale bars: (A and B) 20 μm. (**C**–**F**) Subcellular glycogen distribution in EYFP‐labeled cortical astrocytes were analyzed by two methods; glycogen puncta density (particles/μm^2^; C and D) and area fraction of glycogen immunofluorescence (%; E and F). Blue and red bar graphs represent analyses from IV58B6 and ESG1A9 data, respectively. (mean ± SEM, *N* = 5 animals, *n* = 45 images, *P < 0.05, **P < 0.01, ***P < 0.001, one‐way ANOVA with Tukey HSD test). (**G**–**J**) Subcellular of glycogen distribution analyses in the hippocampus, performed in the same manner as (C–F) (mean ± SEM, *N* = 5 animals, *n* = 47 images, *P < 0.05, **P < 0.01, ***P < 0.001, one‐way ANOVA with Tukey HSD test).

Since GFAP is an intermediate filament protein that delineates the cytoskeletal profile of astrocytes' somata and main branches, the entire astrocytic morphology cannot be evaluated by GFAP staining. To analyze glycogen distribution within individual astrocytes, we expressed EYFP in a sparse population of astrocytes using GLAST‐CreERT × Rosa26‐LSL‐EYFP knockin mice so that the morphology of individual astrocytes is visualized in greater detail. Since the EYFP protein loses its fluorescence after microwave fixation, an antibody designed for denatured GFP (Nakamura et al., 2008) was used to visualize the morphology of the astrocytes. We asked if glycogen is localized in astrocytic somata, primary branches, or higher order fine processes by confocal microscopy (Fig. 4A,B). We observed that somata yielded relatively weak IR for glycogen whereas primary branches and fine processes showed higher IR for both cortical and hippocampal astrocytes. For quantitative analysis, we computed the number of glycogen immunoreactive puncta per 1 µm^2^ area and the immunolabeled area fraction (a portion of glycogen occupying area) for three astrocytic compartments: somata, primary branches, and fine processes. As a result, glycogen puncta were found to be highly localized in processes than somata. Particularly high amounts of glycogen puncta were observed with IV58B6 in fine processes (Fig. 4C,D,G,H). Regarding area fraction, glycogen in primary branches and fine processes showed similar values in the cortex (Fig. 4E,F), while the hippocampus showed the highest area fraction in primary branches with both IV58B6 and ESG1A9 (Fig. 4I,J). The mismatch of glycogen puncta density and area fraction suggested that the immunoreactive puncta size in primary branches is larger than in other compartments. Accordingly, we computed the size distributions of glycogen immunoreactive puncta by astrocytic compartments in the cerebral cortex and hippocampus (Supporting Information Fig. 7). Indeed, glycogen immunoreactive puncta size in primary branches was significantly larger than other compartments. This tendency was particularly evident for hippocampal astrocytes. Since primary branches have the highest area fraction with ESG1A9, a significant portion of large molecule glycogen is considered to be accumulated in clusters in primary processes of hippocampal astrocytes.

### Depth‐dependent Glycogen Distribution in the Cerebral Cortex

**Figure 5:**
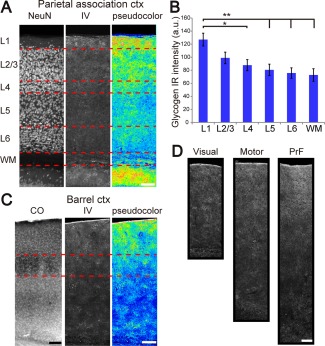
Glycogen distribution in the cerebral cortex. (**A**) IV58B6 glycogen IHC (middle) of the parietal association cortex is displayed with NeuN IHC of the contiguous section for neuronal cell body distribution (left). Pseudocolored image of the IV58B6 labeling is shown on the right. (**B**) Comparison of IV58B6 signal intensity across cortical layers of the parietal association cortex (mean ± SEM, *N* = 9, *P < 0.05, **P < 0.01, ***P < 0.001, one‐way ANOVA with Tukey HSD test). (**C**) Comparison of cytochrome c oxidase staining (CO) (left, brightness and contrast adjusted) and IV58B6 IHC (middle) for the barrel area of the primary somatosensory cortex demonstrates a lack of distinct Layer 4 (between red dotted lines) barrel structure in glycogen distribution. Pseudocolored image of the IV58B6 labeling is shown on the right. (**D**) IV58B6 IR patterns in the primary visual cortex, primary motor cortex, and prefrontal cortex (PrF). There is a general tendency that superficial layers yield higher signals than deep layers, as shown in (B) for the parietal association cortex. Scale bars: (A, C, and D) 100 μm.

The cerebral cortex did not have high amounts of large molecule glycogen, as shown by ESG1A9 IR which yielded weak signals throughout the cortical layers (Figs. 2A,B and 3A). By contrast, confocal imaging with IV58B6 showed a layer‐dependent pattern. An example of IV58B6 glycogen IHC of the prietal association cortex is shown in Fig. 5A. Subsequently, we measured the intensity of IV58B6 immunofluorescence in each layer of the parietal association cortex (Fig. 5B). Layer 1 was distinguished by the highest glycogen IR. Of note, there was a sign of clustered or patchy pattern of glycogen labeling, although the geometrical pattern of the clusters was not well defined. In the rest of the cortical gray matter, glycogen labeling attenuated with cortical depth. The subgriseal cortical white matter had a similar intensity as Layer 6. Other areas of the cortex, for instance, the visual, barrel, motor, and prefrontal areas, showed similar cortical‐depth dependent glycogen labeling patterns (Fig. 5C,D). Since glycogen is related to energy metabolism, we investigated the barrel structure of the primary sensory cortex, which is known to be metabolically highly active consequent to whisker sensation signals. Layer 4 barrels were visualized by cytochrome‐c oxidase staining (Fig. 5C, left column). However, Layer 4 of barrel cortical IV58B6 glycogen IHC appeared similar to Layer 4 of other cortical areas and we could not find obvious barrel structures. Cortical glycogen distribution therefore does not appear to represent the general metabolic demand visualized by cytochrome‐c oxidase staining, implying additional roles of glycogen besides energy substrate.

### Stratified and Patchy Glycogen Distribution in the Hippocampus and Striatum

**Figure 6:**
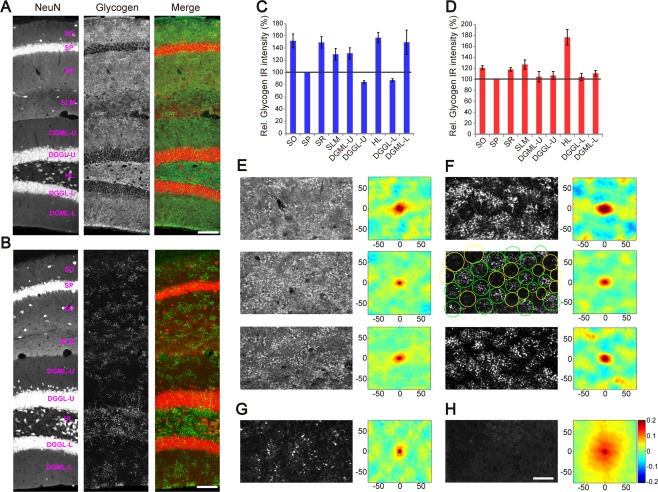
Stratified and patchy distribution of glycogen in the hippocampus. Glycogen IHC by IV58B6 (**A**) and ESG1A9 (**B**) in the hippocampal CA1 and dentate gyrus. In the merged panel (right), NeuN (left) and glycogen (middle) signals are displayed in red and green, respectively. SO *stratum oriens*, SP *stratum pyramidale*, SR *stratum radiatum*, SLM *stratum lacunosum‐moleculare*, DGML‐U dentate gylus molecular layer upper blade, DGGL‐U dentate gylus granular layer upper blade, HL *hilus*, DGML‐L dentate gylus molecular layer lower blade, DGGL‐L dentate gylus granular layer lower blade. Layer‐dependent glycogen IR signal profile by IV58B6 (**C**) and ESG1A9 (**D**). Signal strength of glycogen IHC was quantified (*N* = 5) for various layers of the hippocampus by taking the CA1 pyramidal cell layer as reference (mean ± SEM). (**E** and **F**) Images from CA1 *s.r*. with IV58B6 (left) and ESG1A9 (right) are displayed for autocorrelation analysis. 2D‐autocorrelogram is shown on the right side of each micrograph. High and low correlation areas appear in alternation, indicating patchy distribution. Patchy distribution is more evident with ESG1A9. Images were taken from three individual animals. Green and yellow circles correspond to “on‐patch” and “off‐patch” areas, respectively. (**G**) Patchy glycogen distribution and 2D‐autocorrelogram in the striatum. (**H**) Similar analysis for L2/3 of the cerebral cortex, where patchy distribution is not observed. Images in (A) and (B) are in the same scale. Histological images (upper row) in (E–H) are in the same scale and numbers on the axes of 2D‐autocorrelograms (lower row) are in μm. Scale bars: (A and B) 100 μm; (H) 50 μm.

As the hippocampus is one of the richest regions in glycogen content, we investigated the glycogen distribution in the hippocampus in more detail. We noticed that hippocampal glycogen IR by IV58B6 has a layer‐dependent intensity profile (Fig. 6A). We computed the layer‐by‐layer intensity profile of the hippocampal CA1 and dentate gyrus in Fig. 6C (*N* = 5). CA1 and dentate cell body layers were lower in glycogen IR than other layers, owing to low astrocyte density in these layers (Ogata and Kosaka, 2002). Hippocampal neuropil showed higher intensities than cell body layers, with the highest intensity in the hilus (157 ± 9%). The CA1 stratum lacunosum‐moleculare and the upper blade of the DG granule layer (130 ± 9%, 131 ± 9%) were lower in glycogen IR than the CA1 *strata oriens* (*s.o*.) and *radiatum* (*s.r*.) (151 ± 11%, 149 ± 9%). By contrast, ESG1A9 showed a strikingly different labeling pattern. The hilus was particularly strongly immunoreactive for ESG1A9 (Fig. 6B), while all other hippocampal areas had similar intensities (Fig. 6D).

In addition to the punctate distribution, we noticed that glycogen distributions in the hippocampus showed a peculiar pattern—there were areas of intense labeling with a diameter of approximately 50 µm segregated by areas of low signals of similar size. Moreover, this “patchy” pattern was more evident with ESG1A9 (Fig. 6B), suggesting that large‐molecule glycogen is accumulated in the patches. To quantitatively analyze the patch size and interpatch distance, we computed the 2D‐autocorrelation of the CA1 *s.r*. subjected to IV58B6 and ESG1A9 labeling in Fig. 6E,F, respectively. As in the visual inspection, the 2D‐autocorrelogram showed the characteristic spatial clustering of size between 30 µm and 60 µm.

We also examined the glycogen distribution in the striatum since high amounts of glycogen were visualized by IV58B6. Remarkably, striatal glycogen IR by ESG1A9 also showed the characteristic patchy pattern with an interglycogen‐patch interval of 30–60 µm, similar to the ones seen in the hippocampus (Fig. 6G). In contrast, cerebral cortical layers below Layer 1 did not display patchy glycogen patterns (Fig. 6H).

**Figure 7:**
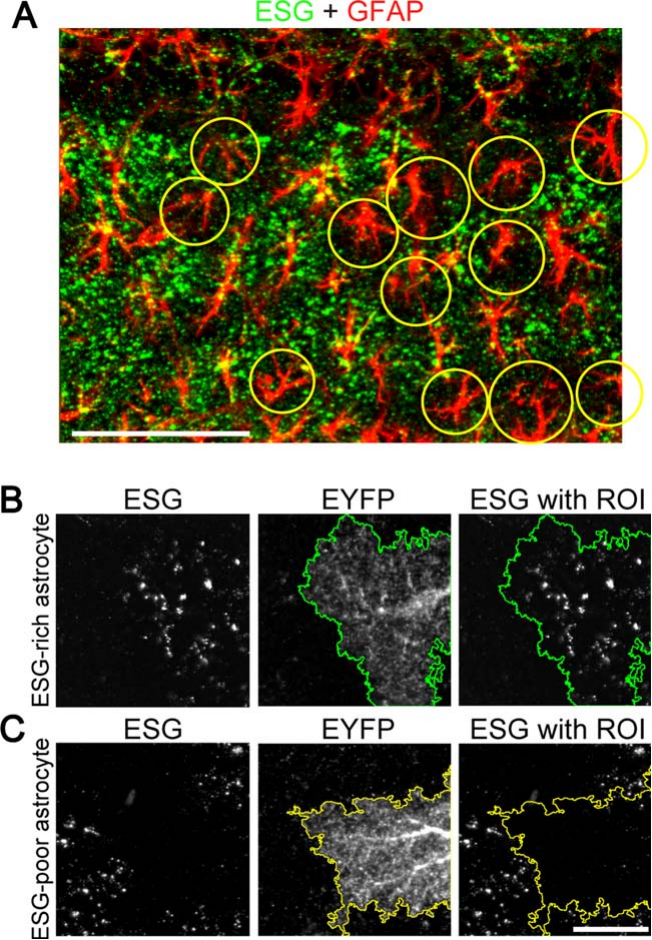
Patchy structure is composed of individual astrocytes. (**A**) ESG1A9 glycogen IHC (green) in the hippocampal CA1 *s.r*. was counterstained with GFAP (red). Some astrocytes are distinctively low in ESG1A9‐labeled glycogen, as marked with yellow circles. (**B**) Example of an ESG1A9‐rich astrocyte. GLAST‐CreERT × Rosa26‐LSL‐EYFP mouse is used to induce EYFP expression in a sparse population of astrocytes. Green line represents the boarder of the EYFP‐expressing and ESG1A9‐rich astrocyte. (**C**) Example an ESG1A9‐poor astrocyte from the same mouse as in (B). Yellow line represents the boarder of the EYFP‐expressing and ESG1A9‐poor astrocyte. Glycogen accumulation depends on individual astrocytes and does not depend on EYFP expression. Images in (B) and (C) are in the same scale. Scale bars: (A) 100 μm and (C) 20 μm.

We next investigated the cellular origin of patchy pattern. To examine the relationship of glycogen patches and astrocyte locations, we performed double IHC of GFAP and ESG1A9‐labeled glycogen in the hippocampal CA1 *s.r*. In addition to colocalization of GFAP and ESG1A9‐labeled glycogen, areas that are devoid of large puncta also followed GFAP pattern (Fig. 7A). This observation proposed that the patchy pattern is regulated by individual astrocytes. To confirm this idea, we asked if ESG1A9‐labeled glycogen is contained within an astrocyte domain by tamoxifen‐induced EYFP expression in sparse astrocytes. As a result, patchy distribution was found to be composed of two types of astrocytes; puncta‐rich astrocytes and puncta‐poor astrocytes. ESG1A9‐labeled glycogen distribution is clearly distinguishable between inside and outside of individual astrocyte domains as shown in Fig. 7B,C. We conclude that individual astrocytes are the unit of the glycogen patch and neighboring astrocytes display heterogeneity in glycogen storage.

### Patchy Glycogen Distribution Diminishes in Aged Mice (1–2 Years Old)

**Figure 8:**
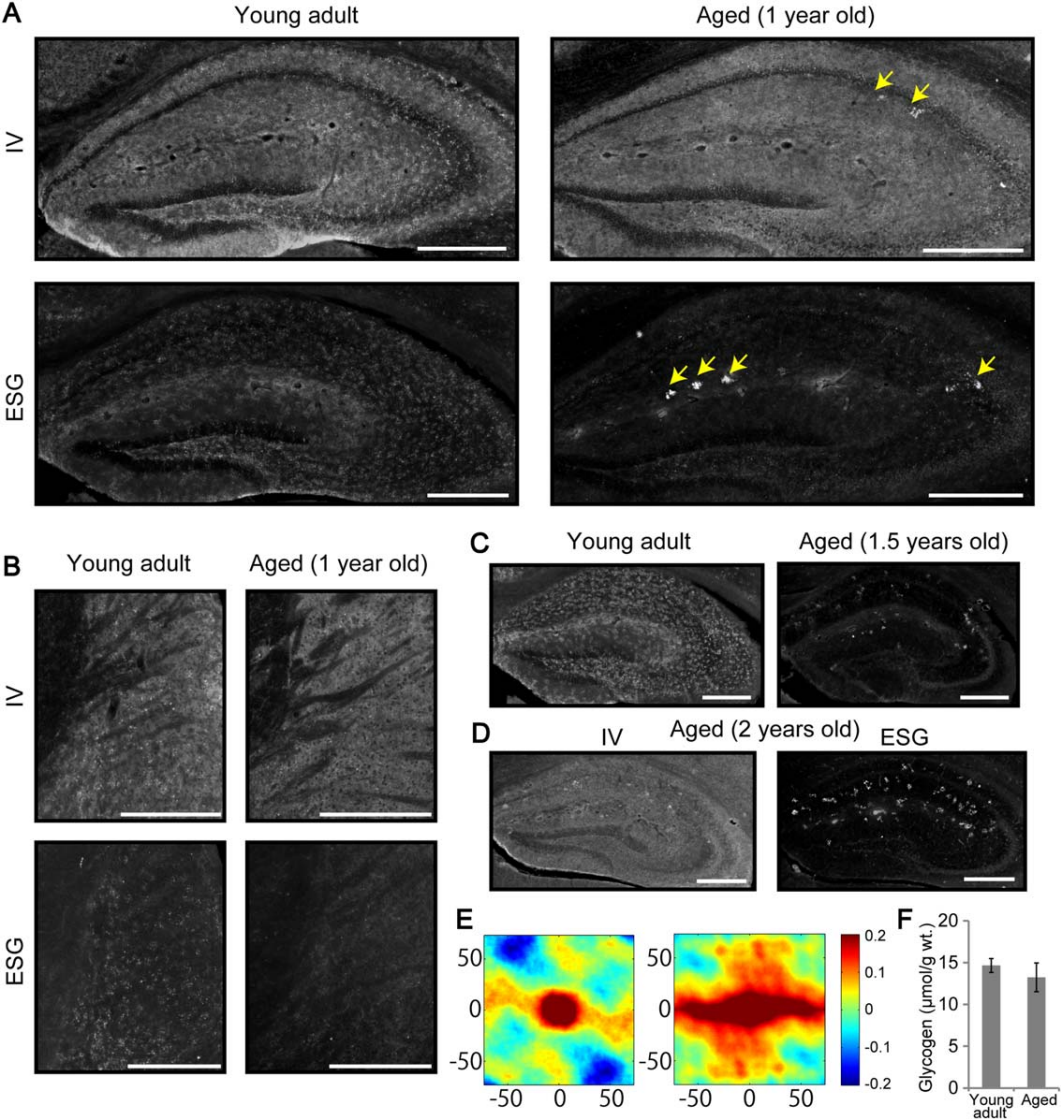
Diminishment of patchy glycogen distribution by aging. (**A**) IV58B6 and ESG1A9 glycogen IHC of the hippocampus from young adult (9–12 weeks old) and aged (1 year old) mice is displayed. Patchy distribution is evident in the young adult mouse whereas IV58B6 signal pattern is smoother and ESG1A9 signal is largely diminished in the aged mouse. Additionally, abnormal glycogen accumulation, known as polyglucosan body, is observable in the aged mouse (arrows). (**B**) Images of the striatal glycogen IHC from the same animals as in (A). ESG1A9 glycogen IHC of the hippocampus from even older mice [(**C**) 1.5 years old and (**D**) 2 years old]. The images from aged mice show clear diminishment of the patchy distribution and more frequent occurrence of polyglucosan with aging. (**E**) 2D‐autocorrelation in the CA1 *s.r*. was computed for a young adult mouse and a 1.5‐year‐old mouse. Numbers on the axes of 2D‐autocorrelograms are in μm. Scale bars: All scale bars are 500 μm. IV58B6 in aged (2 years old) was uniformly stained similar to (A, upper right panel). (**F**) Biochemical assessment of glycogen was performed from young adult and aged (2 years old) mouse hippocampus. In both age groups, similar glycogen amounts were measured. [Color figure can be viewed in the online issue, which is available at wileyonlinelibrary.com.]

In an attempt to understand the biological significance of patchy glycogen distribution, we examined how glycogen distribution is organized in the hippocampus and striatum of aged mice (1 year old). The IV58B6 antibody labeled the hippocampus and striatum with higher intensities than other surrounding structures (Fig. 8A,B), as observed in young adult mice. Importantly, we noticed that IV58B6 IR was more homogeneous in the hippocampus of aged mice, lacking the patchy appearances observable in young adult mice (Fig. 8A). Loss of the patchy glycogen pattern was even more evident with ESG1A9, suggesting that accumulation of large‐fragment glycogen declines in the aged hippocampus. In addition to the homogeneous appearance of glycogen labeling, high‐intensity glycogen clots, called polyglucosan bodies, were present, consistent with previous reports (Cavanagh, 1999, for review). Similar observations were made in the aged striatum in that IV58B6 homogeneously labeled the neuropil well while ESG1A9 was poor in labeling (Fig. 8B). Moreover, such an aging related decline of patchy glycogen pattern and an increase in polyglucosan bodies were more obvious in more aged animals (1.5 and 2 years old, Fig. 8C,D, respectively). We computed 2D autocorrelograms for the hippocampal CA1 *s.r*. of a young adult mouse and an aged mouse (1.5 years old). The autocorrelogram for the aged mouse is distorted because of the strong signals from polyglucosan bodies (Fig. 8E). We noticed that polyglucosan bodies were less frequent in the striatum than the hippocampus of aged mice.

To quantitatively compare the glycogen amount between young adult and aged (2 years old) mice, glycogen assay was performed on the microwave‐fixed hippocampal tissue. There was a small decline in the glycogen level of aged mice, but the difference was not statistically significant (young adult vs. aged, *N* = 4 vs. 3; *P* = 0.453, *t*‐test). These data suggest that an aging‐related transition of glycogen storage could occur by reduction of average glycogen molecule size without affecting the total amount.

## Discussion

We demonstrated that the specificity of glycogen labeling by the IV58B6 and ESG1A9 antibodies is superior to conventional PAS staining. Subsequently, we revealed the distribution of glycogen in the brain by the combination of focused microwave irradiation and glycogen IHC. In agreement with previous work (e.g., Kong et al., 2002; Matsui et al., 2012), we confirm that microwave irradiation is an effective procedure to preserve brain glycogen, which is lost by the process of transcardiac perfusion. The glycogen IR for both antibodies seems to tolerate the postfixation in paraformaldehyde fixative. Since aldehyde‐based fixative cross‐links the proteins at lysine residues, we suppose that the epitopes of the glycogen antibodies are relatively preserved. Since glycogen metabolism has been shown to be crucial in synaptic plasticity and memory formation (Duran et al., 2013; Gibbs et al., 2006; Newman et al., 2011; Suzuki et al., 2011), it is important to understand the distribution of this molecule at macroscopic, cellular, and subcellular levels.

### Astrocytic Localization of Glycogen

Consistent with prior observations in electron microscopy (Cataldo and Broadwell, 1986a, 1986b), we find that glycogen is stored predominantly in astrocytes in the forebrain. In the cerebellum, Bergmann glia are the main glycogen storage. As a common feature between cortical and hippocampal astrocytes, we observed that glycogen is localized primarily in the processes rather than the somata. This result is consistent with a recent study that addressed subcellular localization of glycogen in hippocampal astrocytes using serial block‐face electron microscopy (Calì et al., 2016). According to this study, over a half of observed glycogen particles are found in the vicinity of axon boutons and the rest is distributed near the vasculature and postsynaptic spines. In addition to classical electronmicroscopy work (Maxwell and Kruger, 1965; Koizumi, 1974; Cataldo and Broadwell, 1986a), another recent study by correlative electron microscopy with secondary ion mass‐spectroscopy reported preferential localization of glycogen at astrocytic endfeet in fasted mice injected with ^13^C‐labeled glucose (Takado et al., 2015), hinting at a close association of glycogenesis site with the vasculature. Additionally, we report that astrocytes are the main cells that accumulate glycogen also in the white matter, adding support for influential roles of astrocytic glycogen in action potential conduction in the axons (Brown et al., 2003; Wender et al., 2000). Our analysis showed a small degree of glycogen IR in Iba1 and NeuN positive pixels. However, we should interpret this result carefully. The axial (Z) resolution of confocal microscopy is around 1 µm, a few times larger than the lateral (XY) resolution. It is conceivable that glycogen IR signals in astrocytic fine processes located at slightly different depths are spuriously recognized to colocalized with microglial or neuronal processes (Supporting Information Fig. 6 for possible examples).

Our results show that hippocampal astrocytes accumulate large size glycogen molecules mainly in the primary processes. The functional significance of differentially sized glycogen is not clear (Obel et al., 2012); however, literature in muscle biology suggests that smaller glycogen molecules have higher rates of synthesis (Elsner et al., 2002; Marchand et al., 2007). In the hippocampus, information representation and synapses are reported to change continuously (Attardo et al., 2015; Ziv et al., 2013). In such a condition, the metabolic demand at synapses is conceivably higher than less plastic areas of the brain. It is tempting to hypothesize that the size‐dependent distribution of glycogen reflects the synaptic metabolic demand – smaller glycogen molecules are distributed in perisynaptic fine processes of astrocytes, reflecting the presumed high glycogen turnover.

### Regional Glycogen Distribution and its Potential Roles

As we characterize brain glycogen distribution, we found that distinct brain regions differ in their glycogen amounts. In particular, the hippocampus, superficial layers of the cortex, striatum, thalamic reticular nuclei and cerebellar molecular layer are particularly high in glycogen amount, whereas the hypothalamus and the rest of the thalamus were low. Glycogen has traditionally been regarded as intermediate energy storage. For instance, brain glycogen has been shown to decrease by increased neural activity (Matsui et al., 2012; Swanson et al., 1992). This view would suggest that glycogen‐rich areas are metabolically demanding and the glycogen is readily utilized in instances of high neural activity. Indeed, cerebellar Purkinje cells have high spontaneous firing rates (> 30 Hz) with occasional complex spikes and reticular thalamic neurons fire in bursts during sleep spindles, suggesting high metabolic demands due to the reversal of synapse‐ and action potential‐mediated ion influx.

By contrast, hippocampal principal cells have generally low average firing rates (Csicsvari et al., 1999; Hirase et al., 2001) and low metabolic activity (Gulyas et al., 2006). Cytochrome oxidase staining in this and other studies (Hevner and Wongriley, 1992; Kageyama and Wong‐Riley, 1982) shows intense labeling in CA1 str. l‐m and DG str. mol, which are not particularly outstanding in glycogen labeling. Furthermore, the cytochrome‐oxidase rich whisker barrel structure in the primary somatosensory cortex was not discernible by glycogen IHC. The mismatch between glycogen labeling and cytochrome oxidase histochemistry suggests additional roles of glycogen in brain function.

Remarkably, studies in the recent decade demonstrated the critical role of glycogen‐derived lactate in memory consolidation and LTP‐type synaptic plasticity (Gibbs et al., 2006; Newman et al., 2011; Suzuki et al., 2011). Furthermore, conditional glycogen synthase 1 (GYS1) knockout mice, which lack brain glycogen, were reported to have deficits in operant conditioning task acquisition (Duran et al., 2013). These experiments support the view that lactate shuttle from astrocytes to neurons is a key regulator of synaptic plasticity. Interestingly, lactate has been shown to trigger activation of immediate early genes that are related to synaptic plasticity and enhance the NMDAR current (Yang et al., 2014).

The two forebrain areas with high amounts of glycogen identified in the current study, the hippocampus and striatum, express NMDAR‐dependent synaptic plasticity (Kreitzer and Malenka, 2008; Malenka and Bear, 2004, for reviews) and plasticity in these structures is considered to mediate memory formation and learning. Significant amounts of glycogen are also observed in superficial layers of the cortex, which correlates well with reported adult cortical plasticity in superficial layers, but not in Layer 4 where plasticity diminishes early in postnatal development (Daw et al., 1992; Fox, 1992; Glazewski et al., 1998; Polley et al., 2004). The high glycogen IR signal in cortical Layer 1 could be in part related to the lower neuronal soma density, however, neuron density and glycogen IR are not tightly correlated for other cortical layers (Fig. 5). Together, these observations lend credence to the notion that glycogen metabolism plays a crucial role in synaptic plasticity. Glycogen distribution in disease models with learning or memory deficits will further support the importance of this molecule in synaptic plasticity.

### Patchy Glycogen Patterns in the Hippocampus and Striatum

We observed patchy glycogen patterns in the hippocampus and striatum, in which patches are composed in the unit of single astrocyte morphology. The patchy patterns are apparent because rodent astrocytes are located in a nonoverlapping manner, possessing their own spatial domains (Bushong et al., 2002). Mosaic‐like anatomical structures of various scales have been reported in the forebrain previously (Ichinohe et al., 2003; Maruoka et al., 2011; Ray et al., 2014). These structures are organized by clusters of multiple neurons, reflecting developmental organization or columnar functional coupling. The glycogen patchy pattern observed in the hippocampus and striatum is distinguished from previously observed mosaic patterns in that astrocytes are the principal constituent unit.

The patchy distribution was more pronounced by ESG1A9 which has affinity to large size glycogen. Our observation suggests that glycogen‐rich astrocytes tend to store glycogen by increasing the molecular size of glycogen particles. This could be achieved, for instance, by elevated activity of the glycogen branching enzyme. The patchy distribution of glycogen has not been reported by other gene or molecular markers to our knowledge. Search for glycogen‐patch related genes could lead to a novel definition of astrocyte heterogeneity.

Alternatively, assuming that astrocytes in a given brain region are composed of a relatively homogeneous population (Mishima and Hirase, 2010). The patchy distribution is possibly a reflection of neural activities in the domains of individual astrocytes that affect glycogen consumption or storage. Since glycogenesis and glycogenolysis depends on the cyclic adenosine monophosphate (cAMP) level, activation of G_i_‐ and G_s_‐type G protein‐coupled receptors (GPCRs) may play a role in the emergence of the patchy pattern. Indeed, astrocytes express various functional GPCRs for neuromodulators/transmitters including (nor)adrenergic, cholinergic, dopaminergic, glutamatergic, and GABAergic receptors. It is tempting to speculate that the patches are correlated to a combination of these neuromodulator/transmitter projection and receptor patterns.

The dynamic nature of glycogen particle sizes and their distribution could not be addressed by our immunohistochemical approach. For instance, it remains unknown if glycogen‐rich astrocytes in the patchy pattern remain high in glycogen content throughout their lifespan. Notably, patchy glycogen distribution was not observed in aged mice (1–2 years old), while the glycogen level *per se* did not change significantly. Future studies should address how aging results in the loss of glycogen patchy patterns and how this might be involved in declined memory abilities.

## Author contributions

Conceptualization: Y.O. and H.H. Methodology: Y.O. and H.H. Investigation: Y.O. Writing – original draft: Y.O., H.H., K.C.N., H.A., and O.B. Writing – Review & Editing: Y.O. and H.H. Resources: O.B., H.A., and K.C.N. Supervision: H.H.

## Supporting information

Supporting Information
