# Supplementary material for: Glycogen distribution in the microwave‐fixed mouse brain reveals heterogeneous astrocytic patterns
Source: Glia. 2016 Jun 29;64(9):1532–45. doi: 10.1002/glia.23020 (PMC5094520; doi:10.1002/glia.23020)
Supplement: Supplementary file 1 — Supporting Information [file GLIA-64-1532-s001.docx]

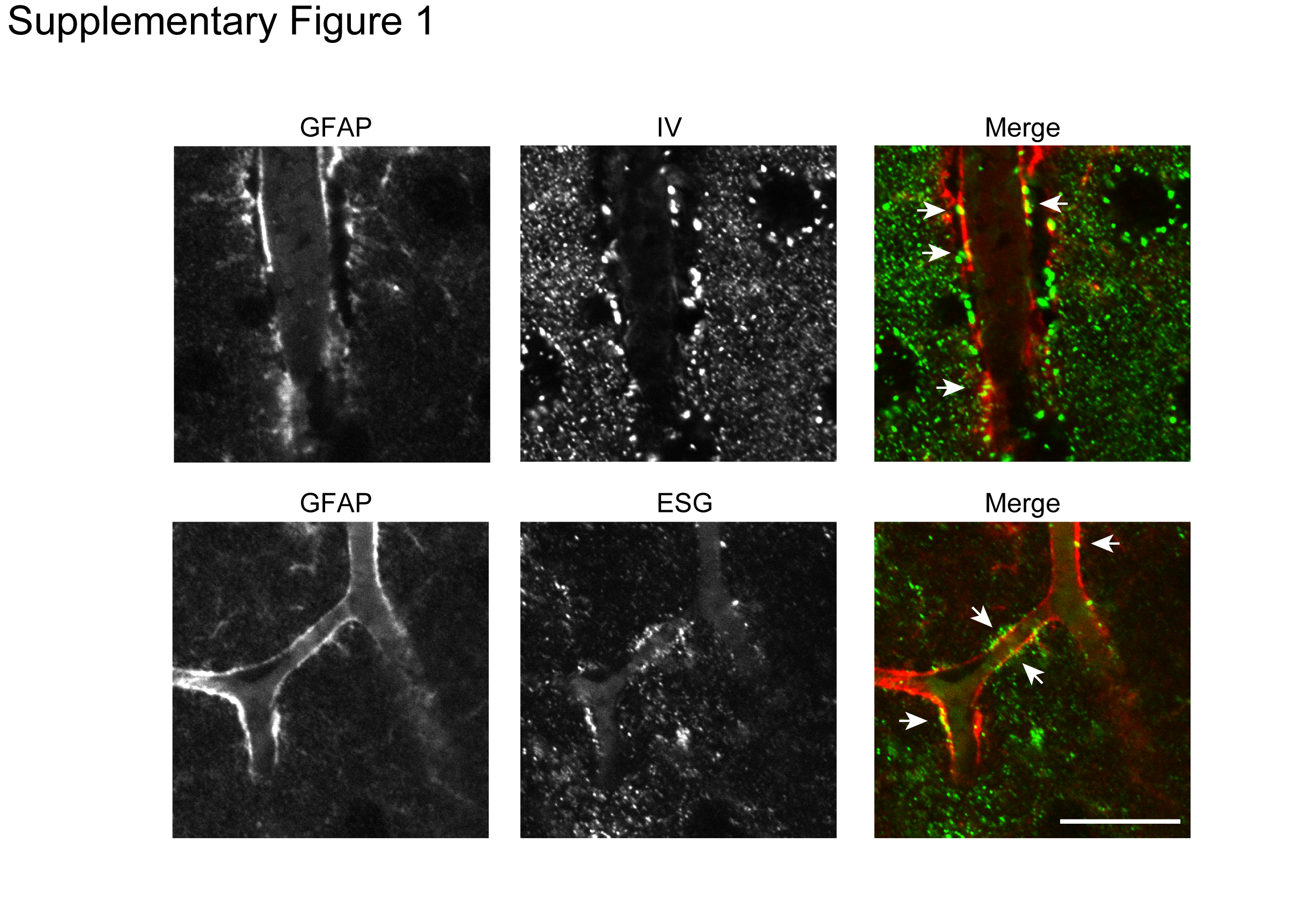


**Supplementary Figure 1.** Representative images of perivascular glycogen IHC in the cortex.

IV58B6 (upper panels) or ESG1A9 (lower panels) glycogen IHC was performed with GFAP IHC. For both IV58B6 and ESG1A9, glycogen IR signals were seen in GFAP endfeet (white arrow). A certain population of endfeet displayed clustered glycogen puncta while other endfeet contained sparsely distributed puncta. Scale bar: 20 μm.


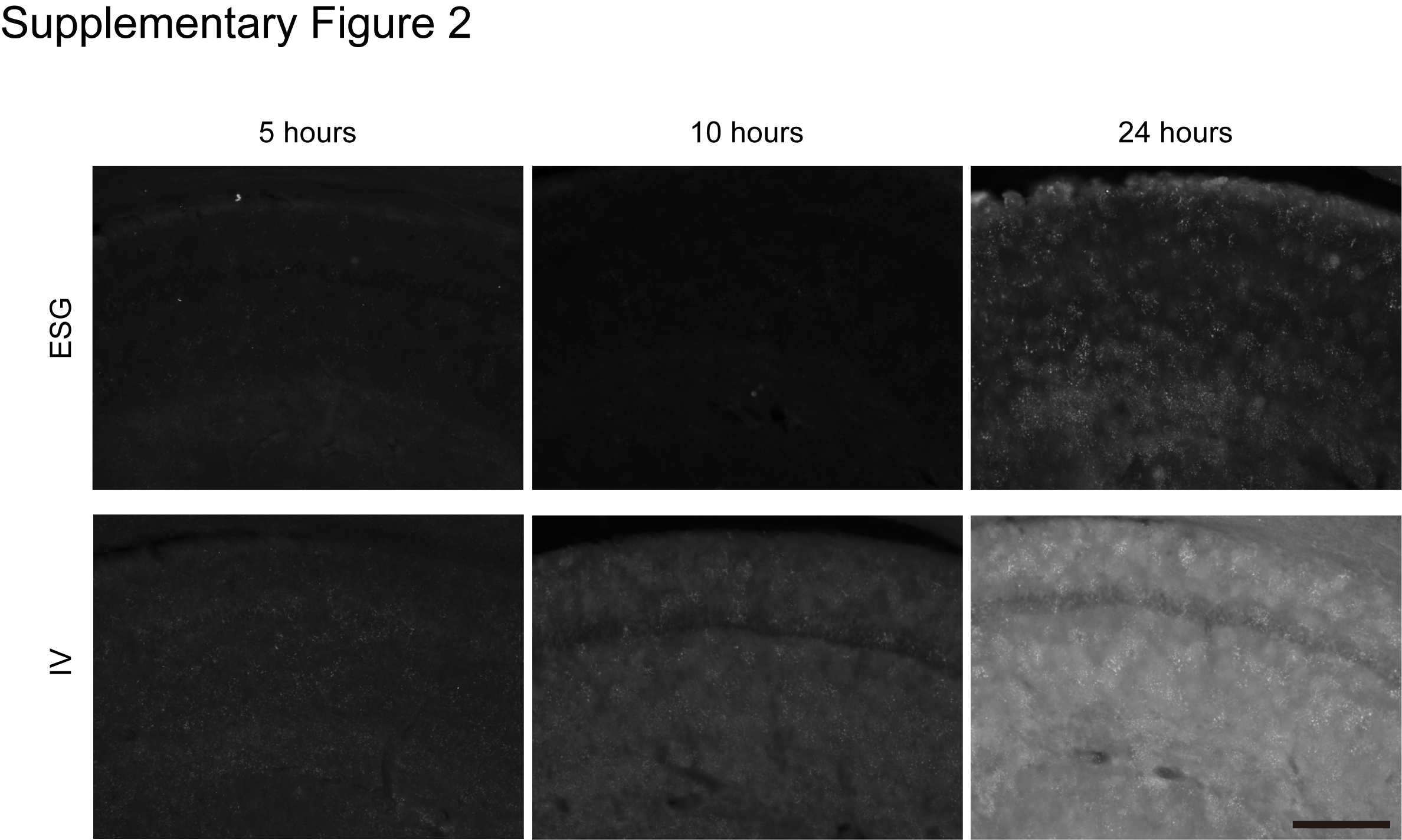


**Supplementary Figure 2.** Evaluation of incubation time for glycogen IHC with IgM antibodies. Incubation periods of 5 hours yielded hardly discernible IR signals (left). Ten hour incubation (equivalent to overnight incubation) showed discernible, but generally low (and sometimes variable) signals. Twenty-four hour incubation yielded sufficient and stable IR signals for both antibodies. Scale bar: 200μm.


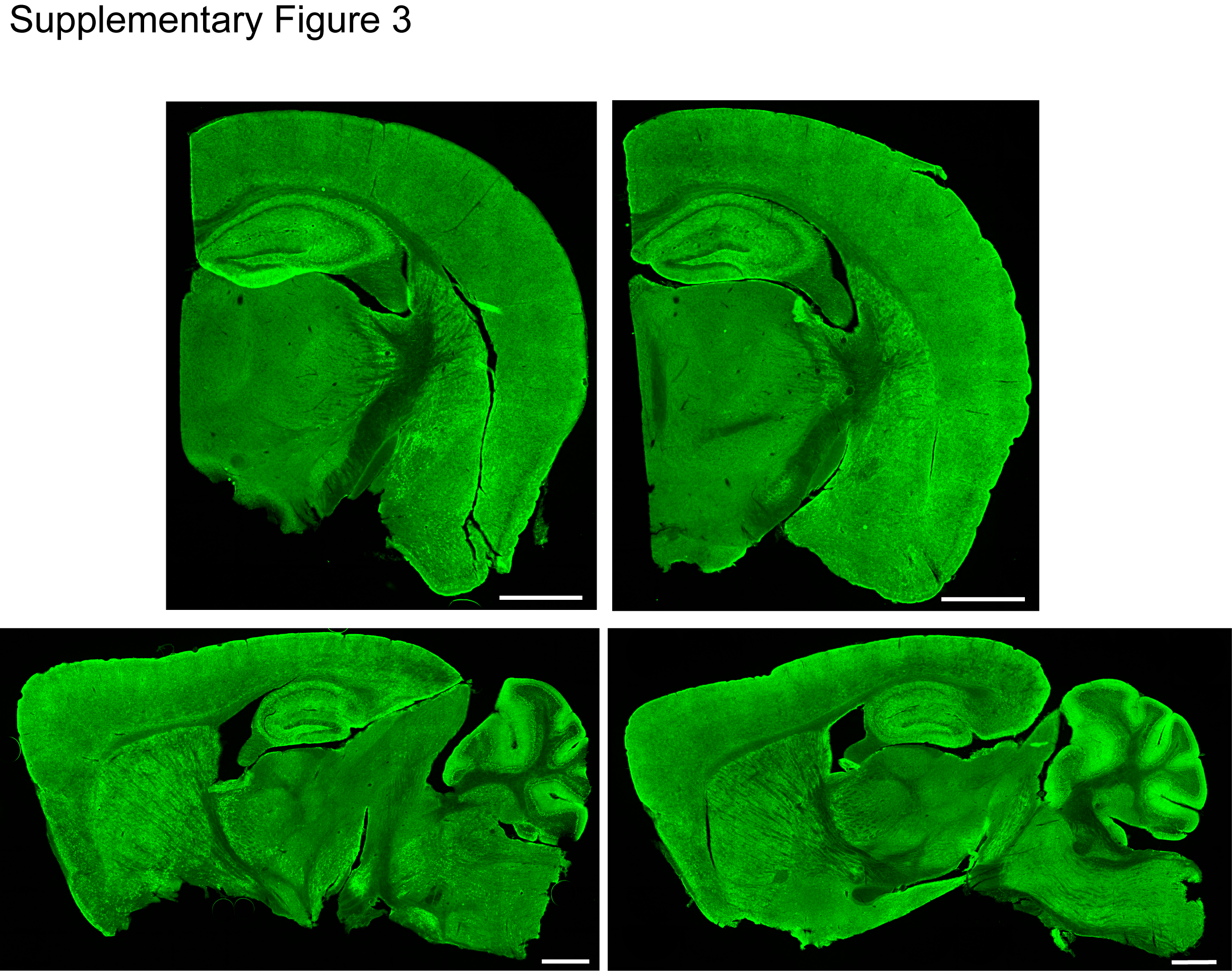


**Supplementary Figure 3.** Examples of IV58B6 IHC. Scale bars: 1 mm.


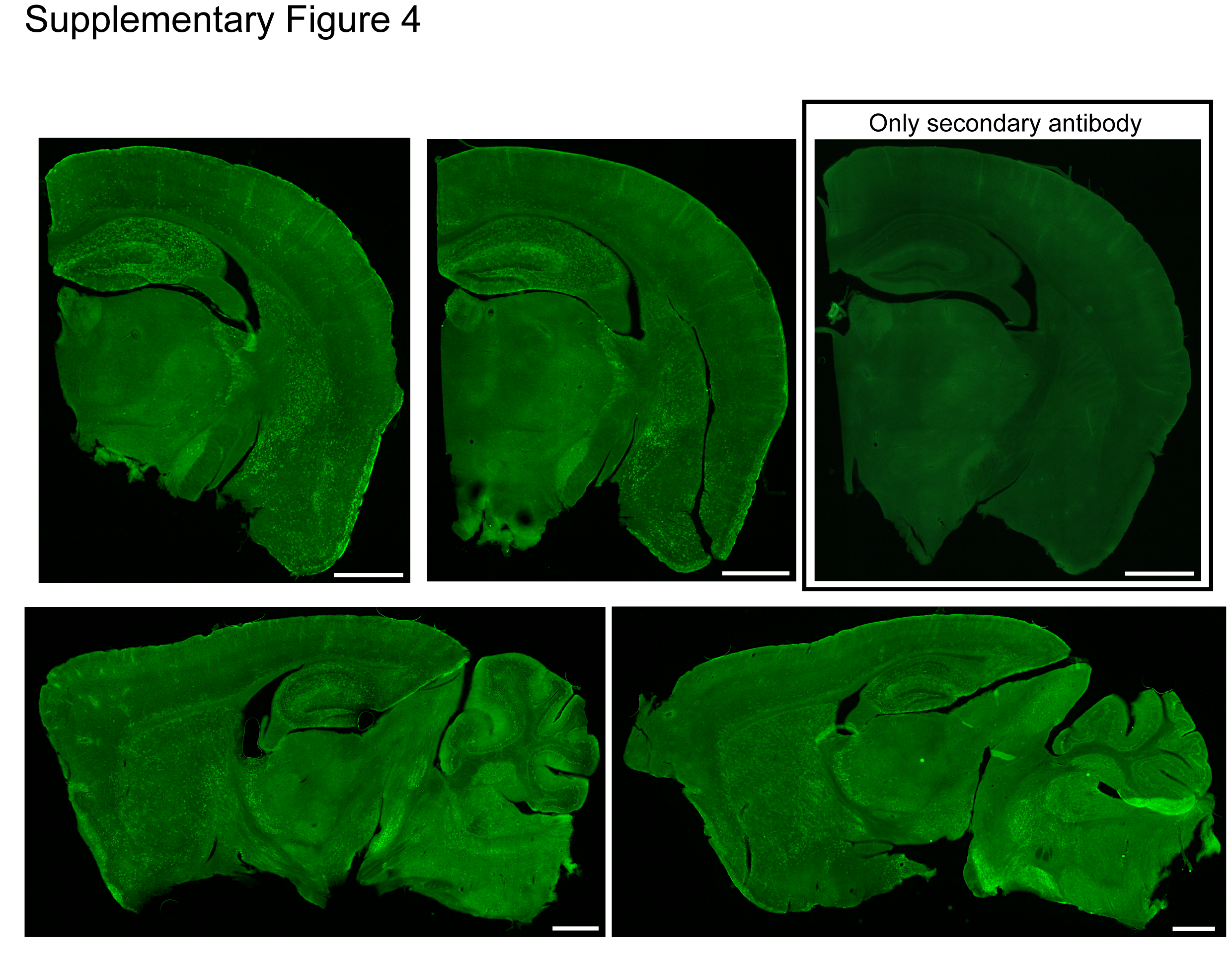


**Supplementary Figure 4.** Examples of ESG1A9 IHC. Control coronal section which was incubated with 0.1% triton X-100 and secondary fluorescent antibody but without primary antibody for 24 hours is displayed in the upper right panel for comparison. Scale bars: 1 mm.


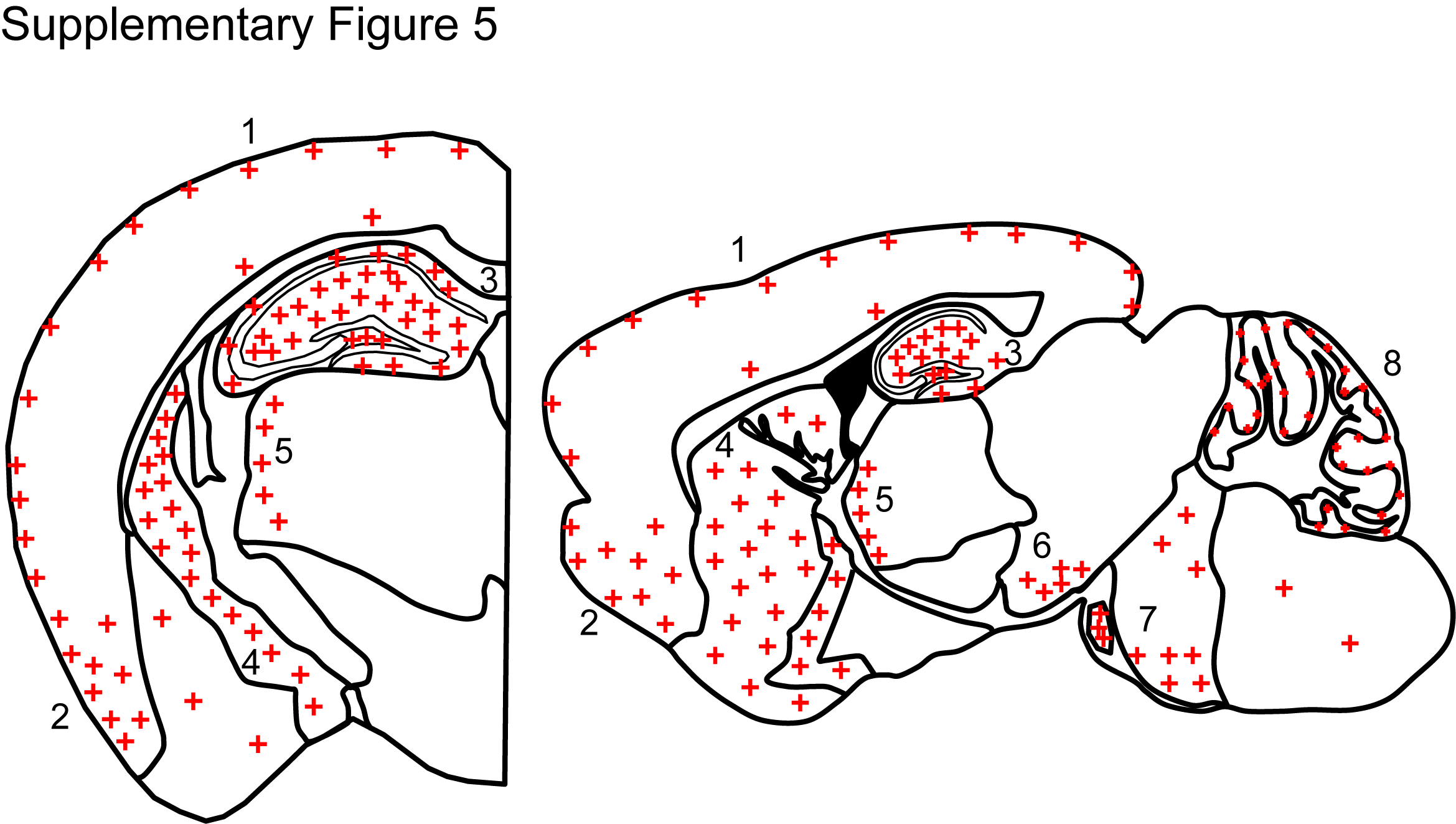


**Supplementary Figure 5.** Qualitative assessment of ESG1A9 IR in coronal and sagittal sections. 1. L1 of the cerebral cortex, 2. piriform cortex, 3. hippocampus, 4. striatum, 5. thalamic reticular nucleus, 6. substantia nigra (pars reticulata), 7. pons (presumably, superior olivary complex, pontine gray, pontine reticular nucleus, lateral lemniscus, trigeminal motor nucleus), 8. molecular layer of the cerebellum.


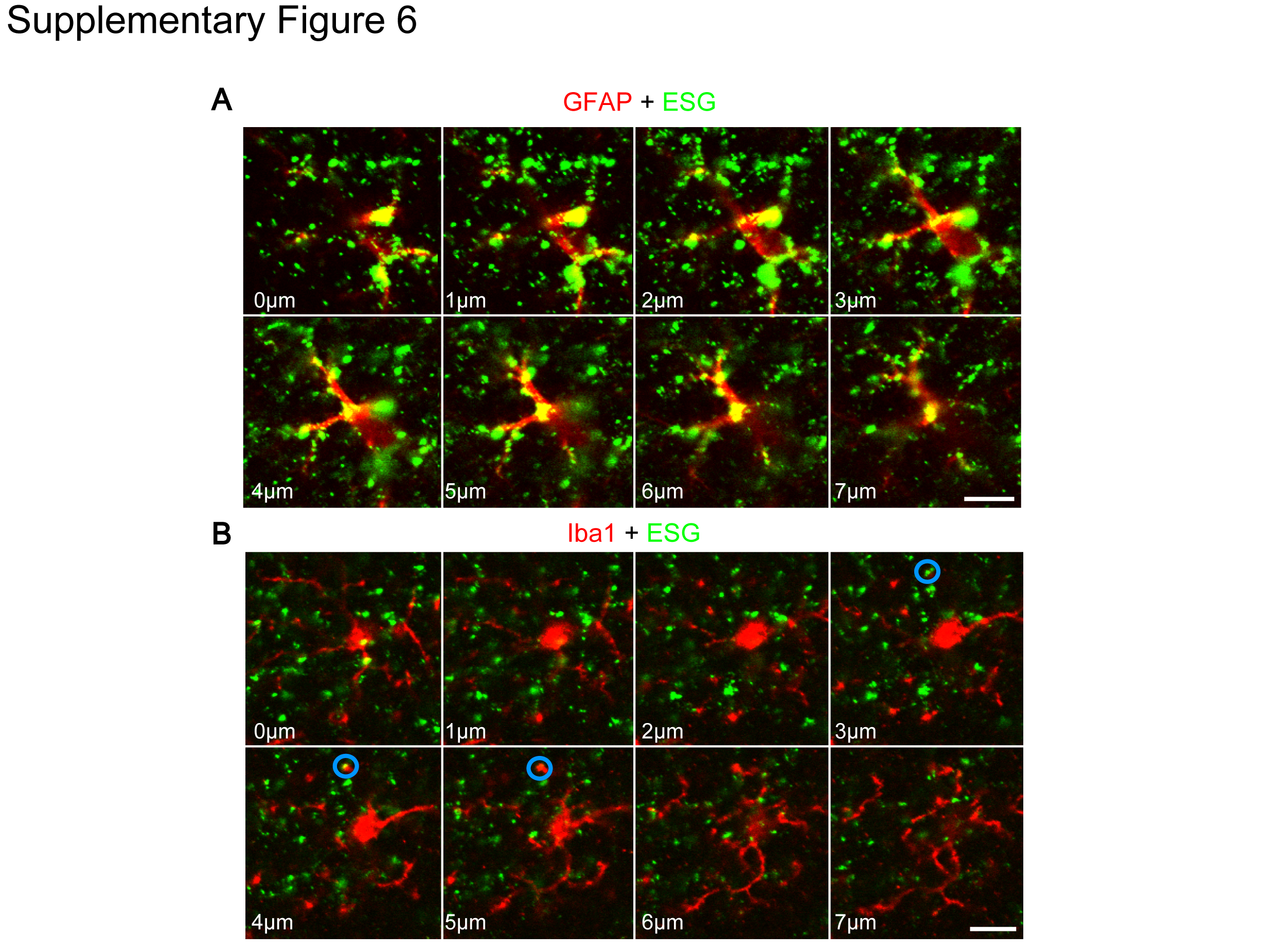


**Supplementary Figure 6.** Sequential depth images taken by confocal microscopy show that glycogen (green) is co-localized with GFAP IR (**A**, red), but hardly with Iba 1 IR (**B**, red). Blue circles mark a possibly spurious Iba 1 positive glycogen signal with ±1 μm depths, showing continuous glycogen IR that span outside the Iba 1 positive region. Scale bar: 20μm.


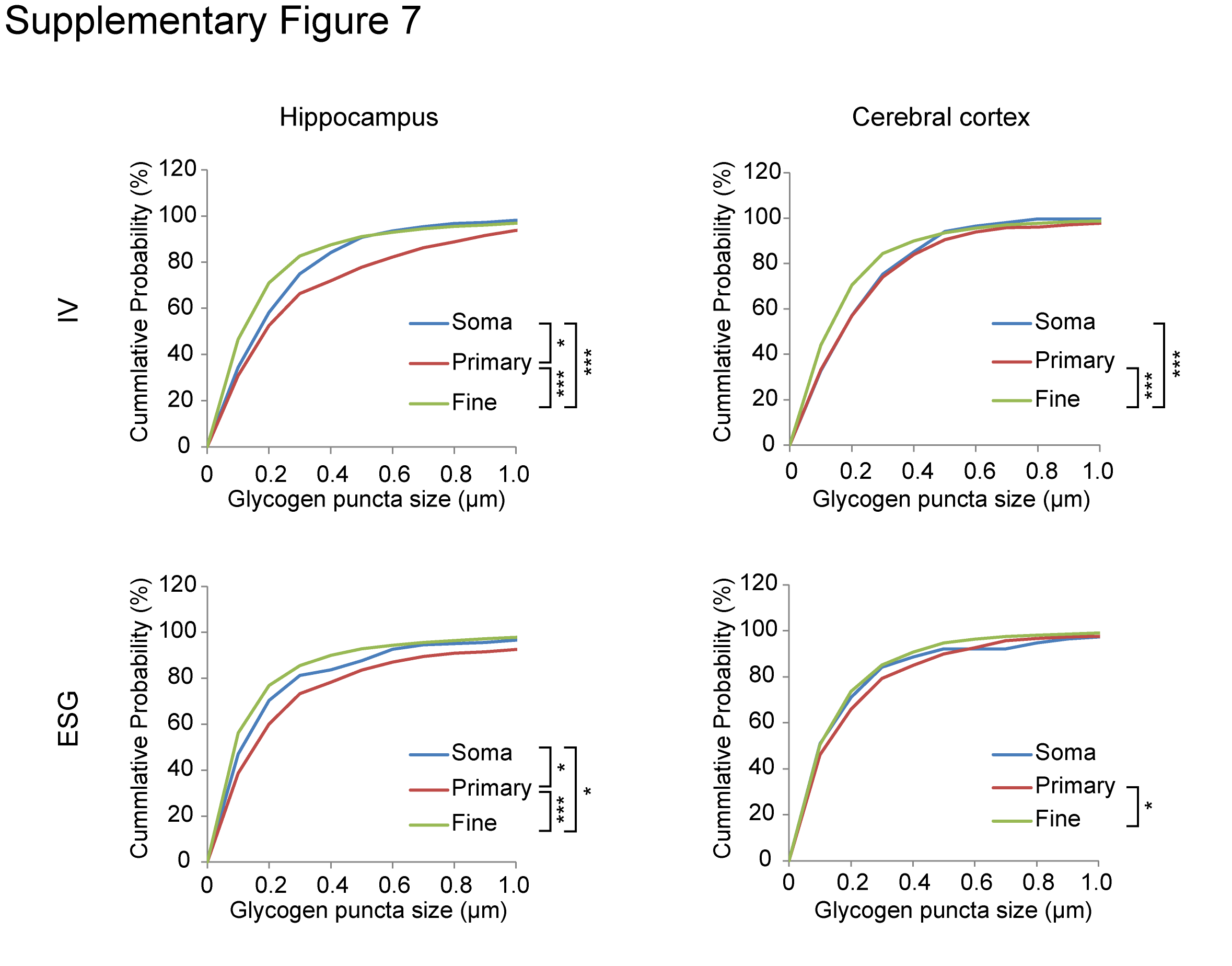


**Supplementary Figure 7.** Distributions of IV58B6 and ESG1A9 IR punctum size. Immunolabeled punctum size distributions are plotted for somata, primary processes, and fine processes of hippocampal (left) and cerebral cortical (right) astrocytes. IV58B6 and ESG1A9 data are plotted in upper and lower panels, respectively. In all cases, glycogen puncta in the primary processes are the largest, particularly in the hippocampus. (mean ± s.e.m, N = 5, *p < 0.05, **p < 0.01, ***p < 0.001, Mann-Whitney U test).
